# Gene Signature Associated with Nervous System in an Experimental Radiation- and Estrogen-Induced Breast Cancer Model

**DOI:** 10.3390/biomedicines11123111

**Published:** 2023-11-22

**Authors:** Gloria M. Calaf, Debasish Roy, Lilian Jara, Francisco Aguayo, Leodan A. Crispin

**Affiliations:** 1 Instituto de Alta Investigación, Universidad de Tarapacá, Arica 1000000, Chile; kcrispi@gestion.uta.cl; 2 Department of Natural Sciences, Hostos College of the City University of New York, Bronx, NY 10451, USA; droy@hostos.cuny.edu; 3 Laboratorio de Genética Humana, Programa de Genética Humana, Instituto de Ciencias Biomédicas (ICBM), Facultad de Medicina, Universidad de Chile, Santiago 8380000, Chile; ljara@uchile.cl; 4 Laboratorio de Oncovirología, Programa de Virología, Instituto de Ciencias Biomédicas (ICBM), Facultad de Medicina, Universidad de Chile, Santiago 8380000, Chile; faguayog@uchile.cl

**Keywords:** axon guidance, neural precursors, neurotransmitter, neuronal markers, breast cancer, radiation

## Abstract

Breast cancer is frequently the most diagnosed female cancer in the world. The experimental studies on cancer seldom focus on the relationship between the central nervous system and cancer. Despite extensive research into the treatment of breast cancer, chemotherapy resistance is an important issue limiting the efficacy of treatment. Novel biomarkers to predict prognosis or sensitivity to chemotherapy are urgently needed. This study examined nervous-system-related genes. The profiling of differentially expressed genes indicated that high-LET radiation, such as that emitted by radon progeny, in the presence of estrogen, induced a cascade of events indicative of tumorigenicity in human breast epithelial cells. Bioinformatic tools allowed us to analyze the genes involved in breast cancer and associated with the nervous system. The results indicated that the gene expression of the *Ephrin A1* gene (*EFNA1)*, the *roundabout guidance receptor 1* (*ROBO1)*, and *the kallikrein-related peptidase 6 (KLK6)* was greater in T2 and A5 than in the A3 cell line; the *LIM domain kinase 2 gene* (*LIMK2)* was greater in T2 than A3 and A5; *the kallikrein-related peptidase 7* (*KLK7), the neuroligin 4 X-linked* gene (*NLGN4X), and myelin basic protein* (*MBP)* were greater than A3 only in T2; and *the neural precursor cell expressed, developmentally down-regulated 9* gene (*NEDD9)* was greater in A5 than in the A3 and E cell lines. Concerning the correlation, it was found a positive correlation between *ESR1* and *EFNA1* in BRCA-LumA patients; with *ROBO1* in BRCA-Basal patients, but this correlation was negative with the *kallikrein-related peptidase 6* (*KLK6)* in BRCA-LumA and –LumB, as well as with *LIMK2* and *ROBO1* in all BRCA. It was also positive with *neuroligin 4 X-linked* (*NLGN4X)* in BRCA-Her2 and BRCA-LumB, and with *MBP* in BRCA-LumA and –LumB, but negative with *KLK7* in all BRCA and BRCA-LumA and *NEDD9* in BRCA-Her2. The differential gene expression levels between the tumor and adjacent tissue indicated that the *ROBO1*, *KLK6*, *LIMK2, KLK7*, *NLGN4X*, *MBP*, and *NEDD9* gene expression levels were higher in normal tissues than in tumors; however, *EFNA1* was higher in the tumor than the normal ones. *EFNA1*, *LIMK2*, *ROBO1*, *KLK6*, *KLK7*, and *MBP* gene expression had a negative ER status, whereas *NEDD9* and *NLGN4X* were not significant concerning ER status. In conclusion, important markers have been analyzed concerning genes related to the nervous system, opening up a new avenue of studies in breast cancer therapy.

## 1. Introduction

Breast cancer is the most prevalent cancer and one of the main causes of cancer death in women, accounting for 685,000 cancer deaths globally in 2020 [1,2]. Chemotherapy resistance is a significant problem affecting the effectiveness of treatment for breast cancer, despite extensive research in the field [3].

Early breast cancer is thought to be treatable if it remains within the breast or has progressed only to the axillary lymph nodes [4]. Nevertheless, a prognosis in the late stages, when cancer has disseminated to distant places, is difficult to treat, despite the advancements in endocrine therapy. A familial connection exists in 10% of cases, and mutations in the tumor suppressor genes BRCA1 and BRCA2 confer a hereditary risk [5]. The genomic amplification of ERBB2, a gene that codes for the epidermal growth factor receptor 2 (HER2), is one of the additional contributing factors [4]. According to studies [6,7,8], humanized anti-HER2 antibodies like trastuzumab and pertuzumab, as well as selective estrogen receptor modulators (SERMs) like tamoxifen and toremifene, represent the gold standard for treating patients with breast cancers that are ER and HER2 positive, respectively.

Until recently, the active interaction between the nervous system and breast cancer cells in the tumor microenvironment was not recognized. The nervous system has long been seen as a bystander in breast cancer [9]. Thus, a study described neurotransmitters and growth factors produced by neurons as signal pathways activators that controlled breast cancer cell proliferation, invasion, metastasis, and treatment resistance [10].

Clinical factors such as tumor size, tumor grade, lymph node status, age, and the immunochemical status of the three tumor receptors—estrogen receptor (ER), progesterone receptor (PR), and HER2—are taken into consideration while deciding on adjuvant therapy. Over the past few decades, breast cancer patients have had an increase in survival thanks to the identification of breast cancer subtypes and relapse risk [4]. However, the role of the nervous system has not been contemplated. On the other hand, the initiation and progression of breast cancer have been associated with the dysregulation of axon guidance molecules (AGMs) in the mammary gland, both through autocrine effects on tumor cells and paracrine effects on endothelial cells that stimulate angiogenesis [11].

For instance, the function of the gene *Ephrin* (*EFNA1*) in development has changed significantly over the past ten years compared to how it was initially described where it served as an attracting or repulsive element to direct axonal growth and migration [12]. It was demonstrated that such a gene serves as a mediator not only to tissue morphogenesis but also cell adhesion and proliferation in the mammary gland [11]. Since then, the functions of the *Ephrin* gene have been expanded to include roles such as regulating tissue morphogenesis and development in a variety of organs, but it can be considered as having potential role in the development of new treatments and diagnostic markers for breast cancer because of such a dual role in carcinogenesis and progression [11].

On the other hand, a conserved ligand-receptor system called slit guidance ligand 2 (Slit2)/roundabout guidance receptor 1 (ROBO1) has a significant impact on the distribution, migration, axon guidance, and branching of neuronal cells. In gliomas, Slit2 and its transmembrane receptor ROBO1 have various distribution patterns; in contrast to ROBO1, which is highly expressed in many grades of gliomas at both the mRNA and protein levels, Slit2 is weakly expressed in pilocytic astrocytoma, fibrillary astrocytoma, and glioblastoma [13].

New putative members of the human kallikrein gene family have been discovered in the last few years. Five additional putative kallikrein genes have been included in this family and are known as *KLK-L2*, *KLK-L3*, *KLK-L4*, *KLK-L5*, and *KLK-L6*. Human kallikrein6 (protease M/zyme/neurosin) was initially identified as a biomarker for ovarian cancer based on its aberrant expression in tumor cells [14].

Neuroligin 4 X-linked gene (*NLGN4X*) encodes a member of the type-B carboxylesterase/lipase protein family. The encoded protein belongs to a family of neuronal cell surface proteins, and members of this family may act as splice-site-specific ligands for beta-neurexins and may be involved in the formation and remodeling of central nervous system synapses. The encoded protein interacts with discs large homolog 4 (DLG4). The neuroligin as Neuroligin 2X (*NLGN2*) is a well-recognized transmembrane scaffolding protein that functions in synapse development and neuronal signal transduction. However, *NLGN 4X* has been found abundantly expressed in breast cancer tissues [15].

The human brain myelin basic protein (*MBP*) is also distributed in the nervous system and extensively in other tissues, where it can be detected in many types of tumor cells, such as neuroglioma, lung cancer, and breast cancer [16].

The neural precursor cell expressed, developmentally downregulated protein 9 (NEDD9) has also been linked to cancer and it is a component of the metastatic signatures of melanoma, breast cancer, glioblastoma, lung cancer, and head and neck squamous cell carcinoma [17]. *NEDD9* overexpression has been associated with a poor prognosis [18]. However, its function in the biology of breast tumors is not known. Authors have examined the function of *NEDD4* expression and have found that it is linked to the progression of breast cancer and serves as a predictor of a poor prognosis [19]. Thus, the Crk-associated substrate family member *NEDD9* is implicated in the adhesion, migration, invasion, and epithelial–mesenchymal transition of cancer cells as E-cadherin [20]. It is known that cellular invasion during the epithelial–mesenchymal transition pathway is largely mediated via E-cadherin [21].

The genes involved in the nervous system have been overlooked. The approaches to study the main causes of treatment resistance in breast cancer have underestimated the role of such genes in clinical outcomes. The characterization of neuronal influence on tumor growth and the identification of new therapy targets will be made easier through the identification of nerve system signatures linked to the treatment response. This study offers nervous-system-related predictive and prognostic markers. A better understanding of the molecular events of such genes might help in developing novel prognostic biomarkers to predict prognosis or sensitivity to chemotherapy. Because experimental studies on cancer rarely concentrate on the relationship between the central nervous system (CNS) and cancer, this work aimed to analyze the main target genes between the CNS and an experimental breast cancer model.

## 2. Materials and Methods

### 2.1. Cell Lines

DMEM/F-12 (1:1) medium supplemented with antibiotics [100 U/mL penicillin, 100 g/mL streptomycin, and 2.5 g/mL amphotericin B (all from Life Technologies, Grand Island, NY, USA)] was used to culture MCF-10F cells, a negative estrogen receptor cell line. Moreover, 0.02 g/mL of epidermal growth factor (Collaborative Research, Bedford, MA, USA), 0.5 g/mL of hydrocortisone (Sigma, St. Louis, MO, USA), and 10 g/mL and 5% equine serum (Biofluids, Rockville, MD, USA) were added [22,23,24,25,26]. The cell lines from the Alpha model included (i) a control cell line (MCF-10F); (ii) an estrogen cell line (E), a negative estrogen receptor cell line; (iii) a malignant cell line (Alpha3), (iv) a malignant and tumorigenic cell line (Alpha5), positive for estrogen receptors, and (v) the Tumor2 cell line, positive for estrogen receptors [23].

### 2.2. Irradiation

Three days before radiation, exponentially expanding MCF-10F cells were plated in 60 mm diameter stainless steel rings with a 6 µm mylar bottom at a density of 3 × 10^5^ cells per ring. At the Columbia University Radiological Research Facilities, cells were exposed to graded doses of 150 KeV/µm ^4^He ions that were accelerated to 4 MeV using the van de Graaff accelerator, as previously mentioned [27]. These high-energy particles have a LET value similar to the radon progeny particles. The MCF-10F cells were subcultured for 12–14 weeks in between doses and either a single or double dose of 30, 60, or 100 cGy of ^4^He ions was administered. Irradiated cultures were subcultured right away to establish growth kinetics and grown further in culture to test for changed phenotypes. At the same time, samples were frozen as future stock. The remaining cells were then subsequently passaged for additional treatment with radiation and sampled for different altered phenotypes. After that, cells were grown with or without estrogen. Cell growth kinetics, anchorage-independent growth, invasiveness, tumorigenicity, and the expression of the BRCA1, BRCA2, and RAD51 proteins were assessed in irradiated cultures.

### 2.3. Alpha-Model

The immortalized human breast epithelial cell line MCF-10F was exposed to low doses of high-linear-energy-transfer (LET) particle radiation (150 keV/m) and subsequent growth in the presence or absence of 17ß-estradiol to create an in vitro experimental breast cancer model. After receiving either a single 60 cGy dose or two 60 cGy doses of alpha particles, the MCF-10F cell line underwent various stages of transformation that included altered morphology, increased cell proliferation compared to the control, anchorage-independent growth, and invasive capability before becoming tumorigenic in nude mice, as previously described [23]. Briefly, the experimental cell lines were: (a) the parental non-irradiated MCF-10F cell line (Control); (b) a non-irradiated MCF-l0F cell line continuously treated with estradiol at 10^−8^ M (E); (c) MCF-10F cells irradiated with a double dose of 60/60 cGy α particles, named Alpha3 (A3), a malignant and non-tumorigenic cell line; (d) MCF-10F cells irradiated with a double dose of 60/60 cGy plus estrogen, named Alpha5 (A5), a tumorigenic cell line; and (e) the Tumor2 cell line (T2) derived from the mammary tumors formed in nude mice after they were injected with the A5 cell line. The A5 and T2 cell lines were tumorigenic in the model and SCID animal, whereas the E and A3 cell lines did not cause mammary tumors. These cell lines were grown either with or without estrogen for up to 10 months.

### 2.4. Preparation of Fluorescence-Labeled Probes for Microarray Analysis

The QIA-Direct-mRNA Isolation kit (Qiagen, Valencia, CA, USA) was used to isolate the poly(A) mRNA from normal, radiation-treated, and estrogen-treated breast cell lines. Following the standard procedure as previously described [28], fluorescent-labeled cDNA was generated from 1 µg of each of these poly(A)mRNAs using oligo dT-primed polymerization and the Superscript II reverse transcriptase kit (Life Technologies, Grand Island, NY, USA) in the presence of either Cy3- or Cy5-labeled dCTP. For analysis, the relevant Cy3- and Cy5-labeled probes were combined, hybridized to the microarray on glass coverslips for 16 h at 65 °C, and then thoroughly washed.

### 2.5. Analysis of Microarray Gene Expression Using the Affymetrix HG-U133A Plus 2.0 GeneChip

The Affymetrix U133A oligonucleotide microarray (Affymetrix, Santa Clara, CA, USA), which comprises 14,500 genes, was used to assess gene expression in the breast cancer model (Alpha-model), which included the cell lines MCF-10F, Estrogen, Alpha3, Alpha5, and Tumor2. The Affymetrix GeneChip Operating Software (GCOS) v1.0 ST, the Genes@Work software platform, and the discovery algorithm SPLASH (structural pattern localization analysis by sequential histograms), with a false discovery rate of 0.05, were used to quantitatively analyze arrays for gene expression [29].

### 2.6. Bioinformatic Gene Expression Analysis and Statistical Analysis

Through its three main components of Immune Association, Cancer Exploration, and Immune Estimation, TIMER2.0 is a web tool that systematically analyzes immune infiltrates across various cancer types. The Cancer Exploration component included the Gene_DE module that provided the differential gene expression between tumor and normal tissues, displaying the distribution of gene expression levels using box plots; the statistical significance computed via the Wilcoxon test was annotated with the number of stars (*: *p* < 0.05, **: *p* < 0.01, ***: *p* < 0.001); the Gene_Corr module provided the correlation between genes with the statistical analysis carried out using Spearman’s test; and the Gene_Outcome module used the Cox proportional hazard model to evaluate the outcome significance of gene expression adjusted via the stage clinical factor [30].

UCSC Xena, another web resource of information, provided the ER status of the genes involved in this study; the statistical significance was computed via a one-way ANOVA test [31]. *p* < 0.05 was considered significant.

## 3. Results

The approach behind this work was to find the marker genes related to the nervous system, and the marker genes were chosen based on data in the literature. Then, gene expression was measured in the cell lines developed in this experimental cancer model, produced from MCF-10F, exposed to LET radiation, and/or incubated in the presence of estradiol and cell lines, derived from a xenograft of irradiated and estradiol-incubated cells, inoculated in nude mice. Among differentially expressed genes (Table 1), analyzed via Affymetrix HG U-133A oligonucleotide microarray, the expression level of preselected genes was assessed and compared between the model cell lines. The correlation between estrogen receptor α *ESR1* and nervous system genes in BRCA patients was estimated using the web platform TIMER2.0, as well as the differential gene expression levels between tumor and normal tissues, and the clinical relevance of gene expression across various breast cancer subtypes was analyzed using the disease stage factor. Gene expression and estrogen receptor status in TCGA breast cancer were provided via the UCSC Xena resource.

### 3.1. Gene Expression Induced via Radiation and Estrogen

**Figure 1:**
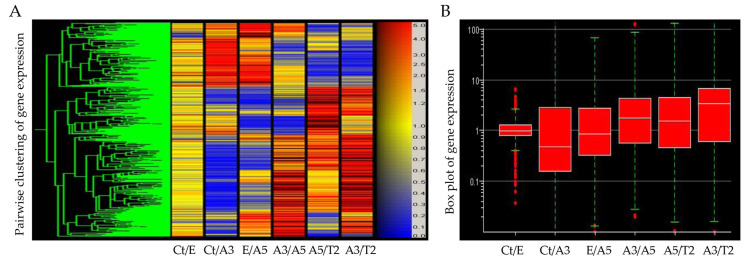
(**A**) Heatmap of Affymetrix array (U133A) data to allow the comparison of the gene expression of the cell lines derived from the model: MCF-10F/estrogen (Ct/E); control/Alpha3 (Ct/A3); estrogen/Alpha5 (E/A5); Alpha3/Alpha5 (A3/A5); Alpha5/Tumor2 (A5/T2); and Alpha3/Tumor2 (A3/T2) (reprinted/adapted with permission from Ref. [28], Spandidos Publications, 2013). The red color indicates a higher expression; blue indicates a lower expression; and yellow indicates an equal expression (left panel). (**B**) The gene box plot summarizes the range of differential gene expression in the same pairwise cell line comparisons (right panel).

High-LET radiation, such as that emitted by radon progeny, in combination with estrogen, caused a series of events that were indicative of cell transformation and tumorigenicity in human breast epithelial cells, according to the profiling of differentially expressed genes obtained using an Affymetrix array U133A (Figure 1).

**Figure 2:**
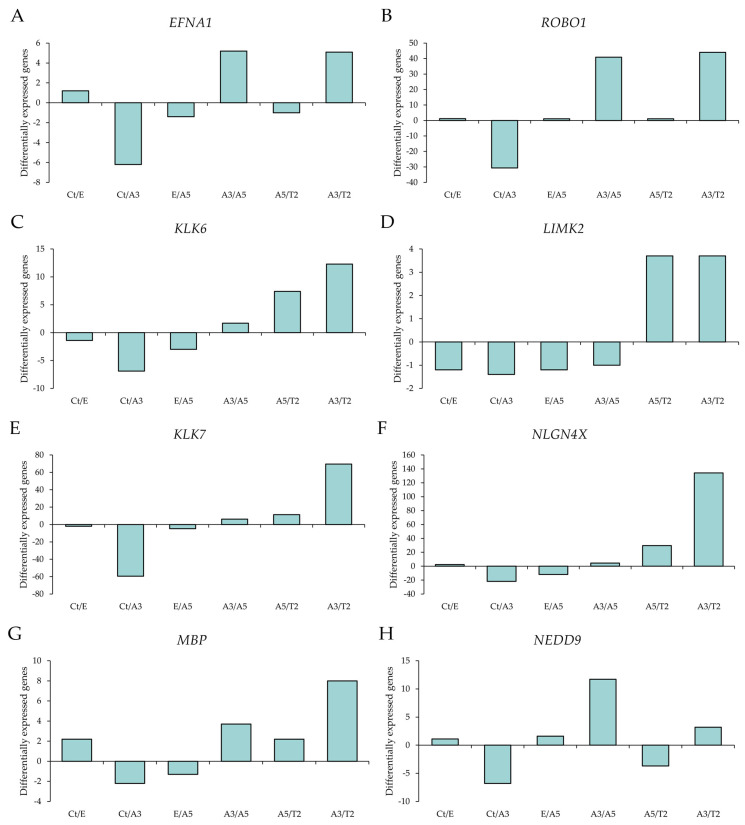
The graphs show the profiling of differentially expressed genes obtained through an Affymetrix array (U133A), with data comparing genes such as (**A**) the ephrin A1 gene, *EFNA1*, (**B**) the roundabout guidance receptor 1 gene, *ROBO1*, (**C**) the kallikrein-related peptidase 6 gene, *KLK6*, (**D**) the LIM domain kinase 2 gene, *LIMK2*, (**E**) the kallikrein-related peptidase 7 gene, *KLK7*, (**F**) the neuroligin 4 X−linked gene, *NLGN4X*, (**G**) the myelin basic protein gene, *MBP*, and (**H**) the neural precursor cell expressed, developmentally down-regulated 9 gene, *NEDD9* in the following cell lines: MCF-10F/Estrogen (Ct/E), Control/Alpha3 (Ct/A3), Estrogen/Alpha5 (E/A5), Alpha3/Alpha5 (A3/A5), Alpha5/Tumor2 (A5/T2), and Alpha3/Tumor2 (A3/T2). The graphs were obtained from a cluster dendrogram repository of gene expression from our laboratory for this article.

The results in Figure 2A,B show that T2 and A5 had greater *EFNA1* and *ROBO1* gene expression than A3. The cell line T2 had greater *KLK6* and *LIMK2* expression than the A3 and A5 cell lines (Figure 2C,D). One of the cell lines, T2, had greater *KLK7, NLGN4X*, and *MBP* expression than A3 (Figure 2E,G) and, finally, the A5 cell line had higher *NEDD9* gene expression than the A3 cell line (Figure 2H).

### 3.2. Correlation between ERα and Nervous System Genes in BRCA Patients

**Figure 3:**
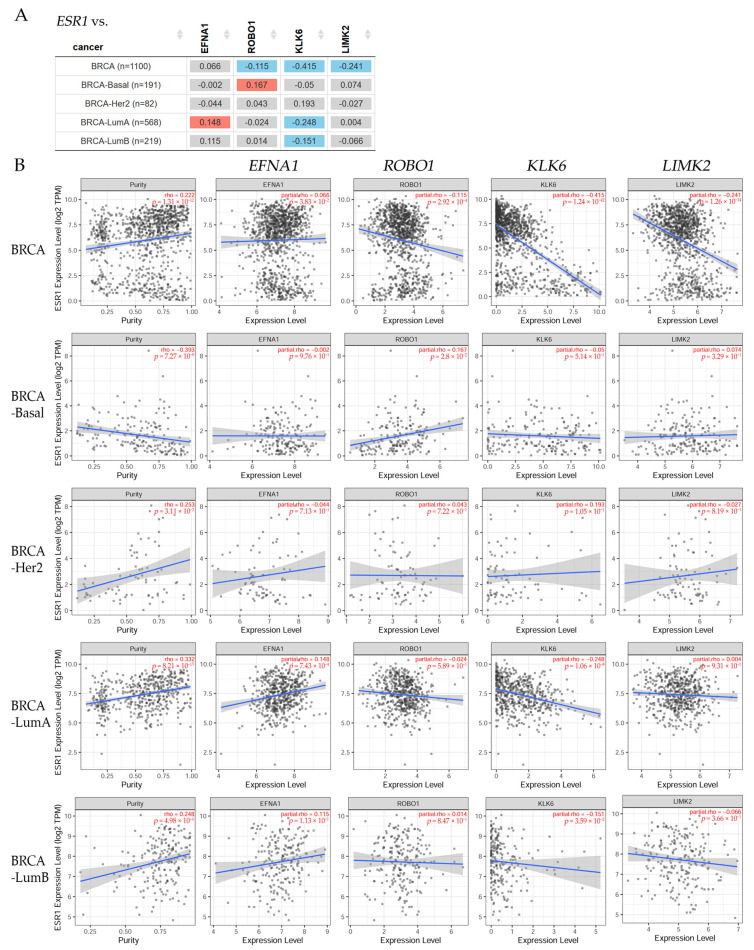
(**A**) Heatmap table and (**B**) box plots representing the correlation between *ESR1* expression with purity adjustment (left) and the ephrin A1 gene, *EFNA1*; the roundabout guidance receptor 1 gene, *ROBO1*; the kallikrein-related peptidase 6 gene, *KLK6,* and the LIM domain kinase 2 gene, *LIMK2* expression levels (right) in breast invasive carcinoma (BRCA) with tumor purity. Heatmap table: the red color indicates a statistically significant positive correlation, the blue color indicates a statistically significant negative correlation, and the gray denotes a non-significant result. Box plots: the correlation values for each analysis are stated in red in the top right of the box. The statistical significance (Spearman’s, *p* < 0.05) was estimated using TIMER2.0, accessed on 17 June 2022 [30].

Figure 3 shows there was a positive significant (*p* < 0.05) correlation between the *ESR1* gene expression level and *EFNA1* expression in BRCA-LumA cancer patients as well as the *ROBO1* expression level in BRCA-Basal patients, but a negative significant (*p* < 0.05) correlation between the *ESR1* and *KLK6* gene expression levels in BRCA-LumA and BRCA-LumB patients, as well as between the *KLK6*, *LIMK2*, and *ROBO1* expression levels in all BRCA patients.

**Figure 4:**
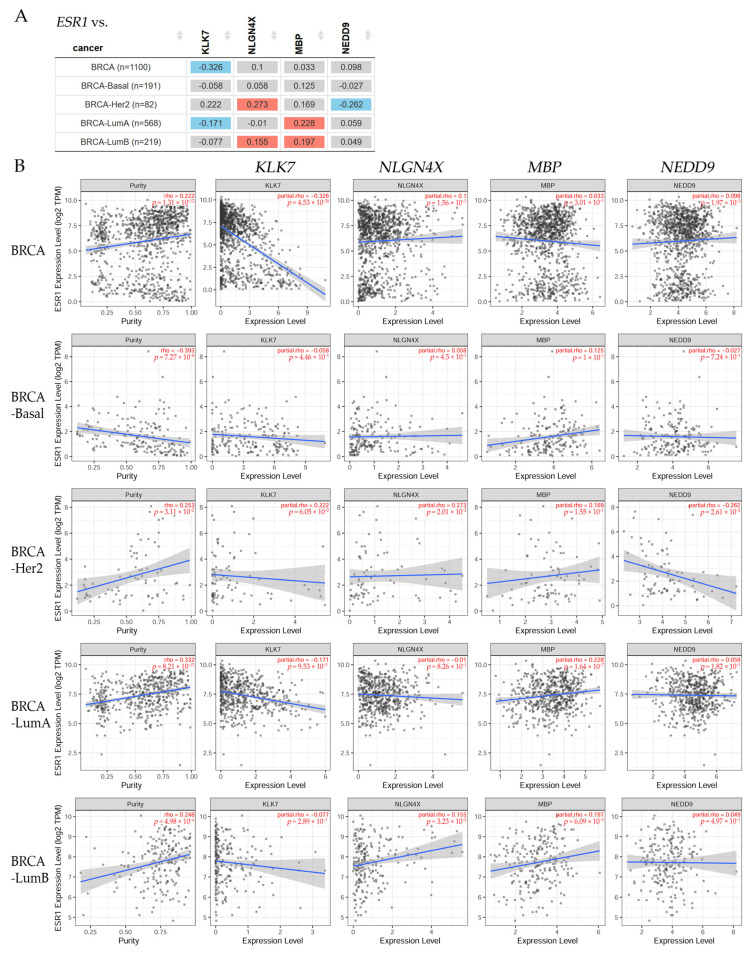
(**A**) Heatmap table and (**B**) box plots representing the correlation between *ESR1* expression with purity adjustment (left) and the kallikrein-related peptidase 7 gene, *KLK7*; the neuroligin 4 X−linked gene, *NLGN4X*; the myelin basic protein gene, *MBP*, and the neural precursor cell expressed, developmentally down-regulated 9 gene, *NEDD9* expression levels (right) in breast invasive carcinoma (BRCA) with tumor purity. Heatmap table: the red color indicates a statistically significant positive correlation, the blue color indicates a statistically significant negative correlation, and the gray denotes a non-significant result. Box plots: the correlation values for each analysis are stated in red in the top right of the box. The statistical significance (Spearman’s, *p* < 0.05) was estimated using TIMER2.0, accessed on 17 June 2022 [30].

Figure 4 shows there was a negative significant (*p* < 0.05) correlation between *ESR1* expression and *KLK7* levels in all BRCA and BRCA-LumA cancer patients as well as in *NEDD9* in BRCA-Her2 breast cancer patients. We also observed a positive significant (*p* < 0.05) correlation between the *ESR1* gene expression level and *NLGN4X* expression in BRCA-Her2 and BRCA-LumB cancer patients and *MBP* expression in BRCA-LumA and -LumB patients.

### 3.3. Differential Gene Expression Levels between Tumor and Normal Tissues across Various Cancer Types

**Figure 5:**
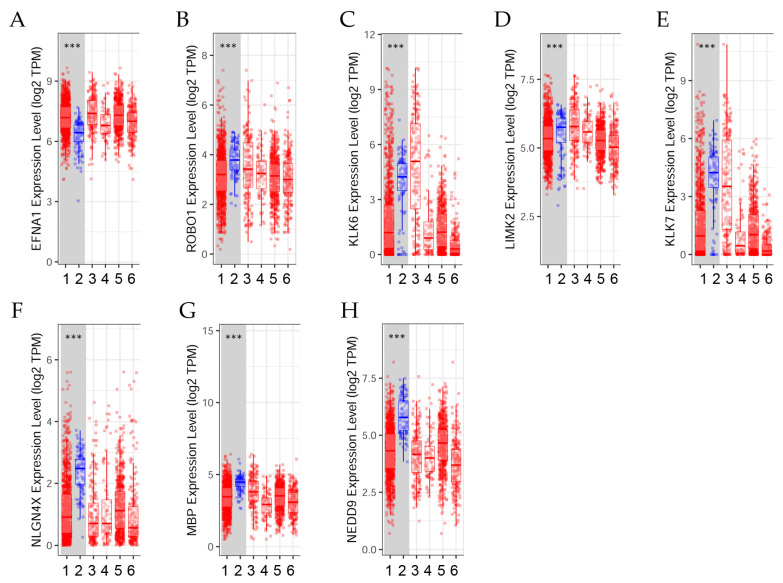
The differential gene expression levels between tumor and normal tissues across various cancer types. The box plots show the distribution of gene expression levels of (**A**) the ephrin A1 gene, *EFNA1*, (**B**) the roundabout guidance receptor 1 gene, *ROBO1* (**C**), the kallikrein-related peptidase 6 gene, *KLK6*, (**D**) the LIM domain kinase 2 gene, *LIMK2*, (**E**) the kallikrein-related peptidase 7 gene, *KLK7*, (**F**) the neuroligin 4 X-linked gene, *NLGN4X*, (**G**) the myelin basic protein gene, *MBP*, and (**H**) the neural precursor cell expressed, developmentally down-regulated 9 gene, *NEDD9* in tumors versus normal tissues (Wilcoxon test, ***: *p* < 0.001) estimated using TIMER2.0 in breast invasive carcinoma, accessed on 17 June 2022 [30]. (1) BRCA.Tumor (*n* = 1093), (2) BRCA.Normal (*n* = 112), (3) BRCA—Basal.Tumor (*n* = 190), (4) BRCA—Her2.Tumor (*n* = 82), (5) BRCA—LumA.Tumor (*n* = 564), (6) BRCA—LumB.Tumor (*n* = 217).

The differential gene expression levels between the tumor and adjacent tissues displayed in the box plot (Figure 5A–H) show that the *EFNA1* expression level was significantly (*p* < 0.001) higher in the tumor tissue than in the normal ones. However, the *ROBO1*, *KLK6*, *LIMK2*, *KLK7*, *NLGN4X*, *MBP*, and *NEDD9* gene expression levels were significantly (*p* < 0.001) higher in normal tissues than in tumors.

### 3.4. Gene Expression and Estrogen Receptor Status in TCGA Breast Cancer

UCSC Xena is an online tool for exploring multiomic, clinical, and phenotypic data [31], including data from The Cancer Genome Atlas (TCGA), which has completed the most thorough cross-cancer investigation to date by creating detailed, multidimensional maps of the main genetic alterations in 33 different forms of cancer [32].

**Figure 6:**
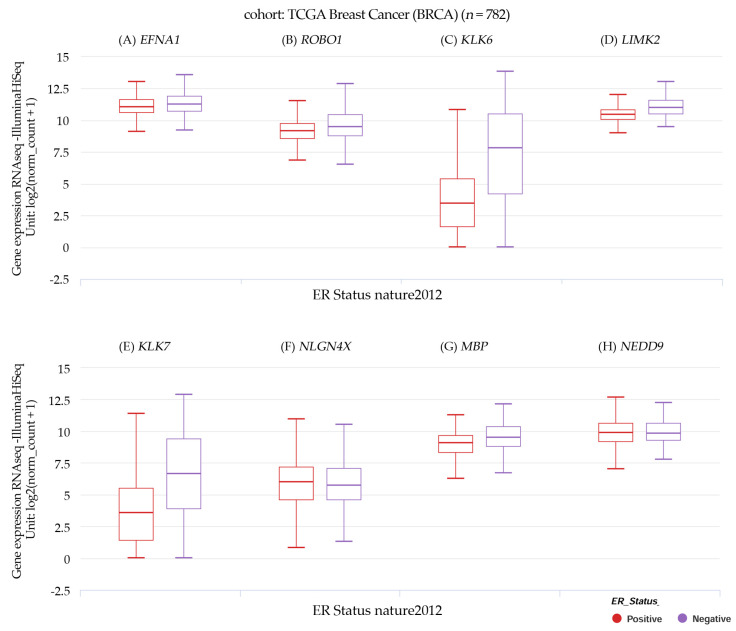
Box plot transcript expression of (**A**) the ephrin A1 gene, *EFNA1*; (**B**) the roundabout guidance receptor 1 gene, *ROBO1*; (**C**) the kallikrein-related peptidase 6 gene, *KLK6*; (**D**) the LIM domain kinase 2 gene, *LIMK2*; (**E**) the kallikrein-related peptidase 7 gene, *KLK7*; (**F**) the neuroligin 4 X-linked gene, *NLGN4X*; (**G**) the myelin basic protein gene, *MBP*; and (**H**) the neural precursor cell expressed, developmentally down-regulated 9 gene, *NEDD9* in breast cancer (TCGA, *n* = 782) stratified by nature2012 for estrogen receptor status (one-way ANOVA, *p* < 0.05). Raw data were extracted from the University of California, Santa Cruz (ena.ucsc.edu). UCSC Xena functional genomics explorer (https://xenabrowser.net) was accessed on 17 June 2022 [31].

The results in Figure 6A–H from the UCSC Xena online exploration tool [31] indicated that the patients’ samples having higher *EFNA1*, *ROBO1*, *KLK6*, *LIMK2*, *KLK7*, and *MBP* gene expression had negative ER, whereas the *NLGN4X* and *NEDD9* gene expressions were not significantly different with regard to ER status.

### 3.5. Disease Stage Factor Analysis of Gene Expression across Various Breast Cancer Subtypes

Numerous genetic analysis studies have shown the molecular components of several pathways, and the discovery of genes linked to particular tissues has helped elucidate their biological role and put disease states like breast cancer, and subtypes like BRCA-Basal, BRCA-Her2, BRCA-LumA, and BRCA-LumB, into context [33,34,35].

The Cox proportional hazard model was used to evaluate the survival difference of gene expression in BRCA (*n* = 1100) patients adjusted by clinical factors. Overall, when the clinical stages of patients were analyzed in breast invasive carcinoma (Table 2), the results indicated that the genes *EFNA1*, *ROBO1*, *KLK6*, *LIMK2*, *KLK7*, *NLGN4X*, *MBP*, and *NEDD9* showed no significance at any stage in BRCA-Basal patients but were significantly (*p* < 0.001) higher in stages 3 and 4 for all BRCA patients and stage 4 for BRCA-LumA patients than in other clinical stages. Additionally, there was a significant (*p* < 0.05 or *p* < 0.01) difference at stage 4 in BRCA-Her2 and BRCA-LumB patients.

## 4. Discussion

The analysis of differentially expressed genes from an Affymetrix array revealed that estrogen and high-LET radiation caused a series of events in human breast epithelial cells that are suggestive of cell transformation and tumorigenicity in the tumorigenic cell lines, where alterations were uncontrolled in comparison to the normal, non-tumorigenic MCF-10F cell line.

Experimental investigations need to focus on the connection between the central nervous system (CNS) and cancer to comprehend the molecular processes required to create new prognostic biomarkers for cancer. Therefore, this study examined the primary CNS-related genes in an experimental breast cancer model.

*EFNA1* had greater expression in T2 and A5 than other cell lines of the model, indicating that the tumorigenic effect induced by radiation and estrogen affected EFNA expression. There was a positive correlation between *ESR1* and *EFNA1* gene expression in BRCA-Lum-A patients, corroborated via the *EFNA1* differential gene expression level being higher in the tumor than the normal tissues and a negative ER status as well as significantly higher EFNA levels in stages 3 and 4 in all BRCA patients and stage 4 in BRCA-LumA patients, and a significant difference in stage 4 in BRCA-Her2 and BRCA-LumB patients, but not significance in any stage in BRCA-Basal patients.

*ROBO1* had a greater gene expression level in the A5 and T2 cell lines than A3, as with the previously mentioned genes. There was a negative correlation between *ESR1* and ROBO1 in all BRCA, but positive in BRCA-Basal patients, indicating that *ROBO1* could be a good complementary gene as a biomarker for breast cancer since there are few markers for BRCA-Basal cancer patients. Patients’ samples with a high *ROBO1* gene expression had a negative ER status, and the differential gene expression levels showed that *ROBO1* expression levels were significantly higher in normal tissues than in tumors. However, *ROBO1* gene expression levels were higher in stages 3 and 4 in all BRCA patients, and in stage 4 in BRCA-LumA patients, than in other clinical stages, and also showed a significant difference in stage 4 in BRCA-Her2 and BRCA-LumB patients. However, this gene showed no significance in any stage in BRCA-Basal patients.

*LIMK2* showed greater expression in T2 than A5 and A3 cell lines of the model, demonstrating that it is a good marker for carcinogenicity. A negative correlation was observed between *ESR1* and *LIMK2* in all BRCA patients. Furthermore, the presence of a positive correlation in BRCA-Basal patients indicates that it can be a good marker for patients who are negative for the present therapy options, such as hormonal responses. The *LIMK2* differential gene expression levels were significantly higher in normal tissues than in tumors, demonstrating that it is a possible tumor suppressor gene. However, other results have shown that *LIMK2* was overexpressed in triple-negative breast cancer (TNBC) and was necessary for facilitating metastasis. To understand the role of *LIMK2* in breast cancer, we first asked whether LIMK2 was overexpressed in breast cancer. Authors [36] analyzed a breast cancer tissue microarray that included 100 cases of invasive breast cancer and 10 adjacent normal breast tissues with information on ER, PR, and HER2 status. They observed that patients’ samples with high *LIMK2* gene expression had a negative ER status, which was also common in advanced stages of this disease.

The present work indicates that *LIMK2* gene expression levels were very high in stages 3 and 4 for all BRCA patients, and stage 4 for BRCA-LumA patients, compared to other clinical stages. Moreover, stage 4 showed a significant difference in patients with BRCA-Her2 and BRCA-LumB. However, in BRCA-Basal patients, this gene displayed no significance at any stage.

A possible target for glioma prevention and treatment is Slit2/ROBO1 signaling. In humans, 4% to 50% of cancers of the nervous system are gliomas, which develop from neural mesenchymal cells [37]. The infiltrative nature of gliomas, which leads to inadequate surgical excision, is one of the main barriers to their effective treatment [38]. Authors reported that malignant glioma cells developed insidious invasiveness through a variety of genetic changes to signaling pathways [39]. It was demonstrated that Slit2/ROBO1 signaling prevented glioma cell motility and invasion by inactivating Cdc42-GTP, even though the precise mechanisms behind this tumor-suppressive gene impact of Slit2/ROBO1 remain unknown [13].

*KLK6* and *KLK7* showed greater expression in T2 than in the A3 and A5 cell lines in the model. There was a negative correlation between *ESR1* and *KLK6* in all BRCA, BRCA-LumA, and LumB patients, and *KLK7* in all BRCA and BRCA-LumA. The differential gene expression levels between the tumor and adjacent tissue showed that *KLK6* and *KLK7* expression levels were higher in normal tissues than in tumors. According to a study [40], the value of the kallikrein-related peptidase 5 gene *KLK5* and *KLK7* in the diagnosis and prognosis prediction of breast cancer patients was far from clear. To further determine their role and clinical significance in breast cancer and to explore the relationship between *KLK5* and *KLK7*, the mRNA levels of *KLK5* and *KLK7* in normal breast tissues, benign breast tissues, primary tumors, and lymph node metastases were detected using a real-time reverse transcription polymerase chain reaction and microarray. The relationship between *KLK5* and *KLK7* expression and clinicopathological parameters and the correlation between the mRNA levels of *KLK5* and *KLK7*, as well as the 5′-noncoding regions of *KLK5* and *KLK7*, were analyzed. The mRNA levels of both *KLK5* and *KLK7* were downregulated in breast cancers relative to normal and benign tissues, and downregulated in metastases compared to primary cancers.

The present study shows that patient samples with higher *KLK6* and *KLK7* gene expression had a negative ER status. *KLK6* and *KLK7* gene expression levels were higher in stages 3 and 4 for all BRCA patients, and in stage 4 for BRCA-LumA patients, than in other clinical stages. Additionally, stage 4 showed a significant difference in patients with BRCA-Her2 and BRCA-LumB. However, in BRCA-Basal patients, this gene displayed no significance at any stage.

Experimental data suggest that *KLK6* may have a potential function in the CNS; the human kallikrein gene family includes kallikrein 6 (hK6), also called *KLK6* (zyme/protease M/neurosin), and other related proteins [41]. Zyme appears to play a role in breast cancer, and the cDNA for the *KLK6* gene has just been discovered [42]. A study [43] indicated that differential proteomic profiling revealed that *KLK6* re-expression resulted in significant down-regulation of vimentin, which represented an established marker of epithelial-to-mesenchymal transition of tumor cells and the concomitant up-regulation of calreticulin and epithelial markers cytokeratin 8 and 19, indicating that *KLK6* might play a protective role against tumor progression that was likely mediated via the inhibition of epithelial-to-mesenchymal transition. Such a study suggested that *KLK6* was an epigenetically regulated tumor suppressor in human breast cancer and provided methods for pharmacologic modulation. The overexpression of the mRNA in breast and ovarian primary cells was demonstrated by authors who earlier cloned the cDNA for this gene via differential display [44].

The *NLGN*4X gene expression level showed greater expression in T2 than the A3 and A5 cell lines of the model. There was a positive correlation between *ESR1* and the gene expression of *NLGN4X* in BRCA Her2 and BRCA-LumB patients. Authors [15] showed that neuroligins were neural cell adhesion molecules that were implicated in hetero-topic cell adhesion and were highly expressed in blood vessels and implicated in the growth of glioma cells; they reported an increased expression of *NLGN4X* in breast cancer tissues. However, our analysis indicated that the differential gene expression of *NLGN4X* levels was higher in normal tissues than in tumors, indicating that other factors could be involved in such a difference. On the other hand, *NLGN4X* expression levels were also higher in stages 3 and 4 for all BRCA patients, as well as in stage 4 for BRCA-LumA patients, compared to other clinical stages. Additionally, stage 4 showed a substantial difference between patients with BRCA-Her2 and with BRCA-LumB. However, in BRCA-Basal patients, this gene displayed no significance at any stage. Furthermore, this gene expression was not significant concerning ER status. It has been reported that the NLGN gene family has great importance in mediating synapse formation in the central nervous system and displays a strong and selective synapse formation, which promotes activity between neurons in vitro.

Even though the *MBP* gene expression was greater in T2 than in the A3 and A5 cell lines of the model, the differential gene expression levels between the tumor and adjacent normal tissue showed that *MBP* expression levels were significantly higher in normal tissues than in tumors, indicating that T2 was greater due to another factor not present in the normal tissues.

On the other hand, the *ESR1* and *MBP* correlation was positive in BRCA-LumA and Lum-B cancer patients. Patient samples with higher *MBP* gene expression had a negative ER status corroborated via higher *MBP* expression levels in stages 3 and 4 for all BRCA patients, as well as in stage 4 for BRCA-LumA patients, than in other clinical stages. Moreover, stage 4 showed a significant difference between patients with BRCA-Her2 and with BRCA-LumB. But, in BRCA-Basal patients, this gene displayed no significance at any stage.

Studies have shown that *MBP* plays a role in immunity: it was serially measured in 177 CSF samples from 33 patients with leptomeningeal metastases and compared to 34 cancer controls to determine the degree of autosensitization to *MBP* [45].

The *NEDD9* gene expression level showed greater expression in the A5 than the E cell line of the model, indicating that radiation was involved in the transformation process. The differential gene expression levels showed that *NEDD9* expression levels were significantly higher in normal tissues than in tumors, corroborating that NEDD9 gene expression is activated before tumor formation. Otherwise, T2 would have been higher than the A5 cell line. A negative correlation was observed between *ESR1* and *NEDD9* in BRCA-Her2 patients. This gene expression was not significant concerning ER status. However, the gene expression levels were significantly different at stage 4 for BRCA-Her2 and BRCA-LumB patients but, for BRCA-Basal patients, this gene showed no significant difference at any stage.

**Scheme 1:**
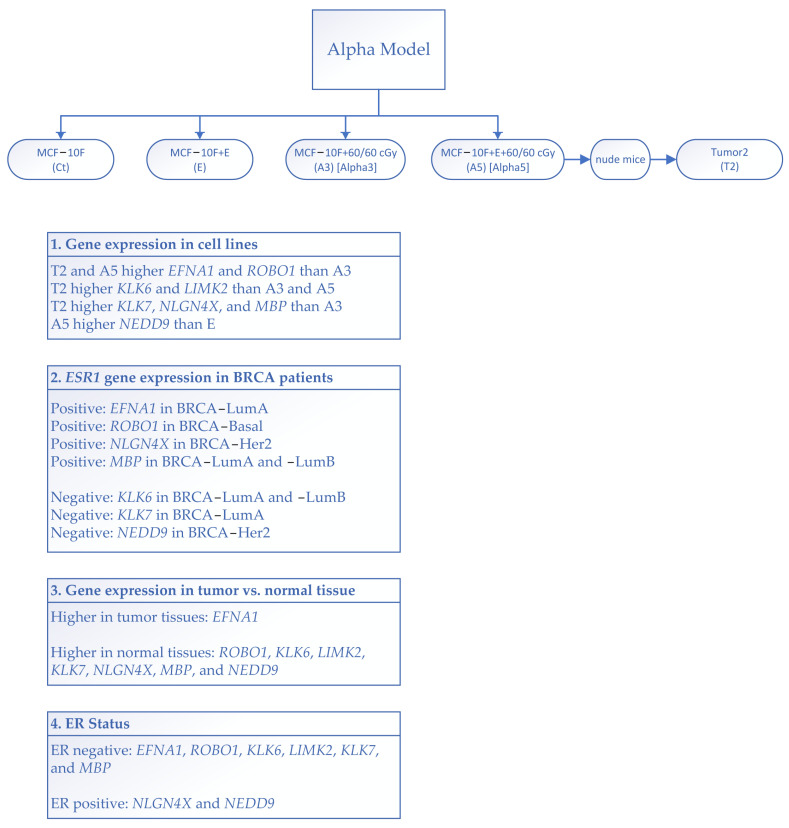
Summary of the alpha model and main findings observed in this study. (1) *EFNA1*, *ROBO1*, *KLK6*, *KLK7*, *LIMK2*, *NLGN4X*, *MBP*, and *NEDD9* gene expression in the estrogen (E), the Alpha3 (A3), the Alpha5, and the Tumor2 (T2) cell lines; (2) estrogen receptor α gene *ESR1* expression in BRCA patients; (3) differential gene expression between tumor and normal tissues; (4) estrogen receptor status.

The T2 cell line had greater *EFNA1*, *ROBO1, KLK7, NLGN4X,* and *MBP* gene expression when compared to A3, a non-malignant cell line treated with alpha particles without estrogen, or the A5, treated with both. Such results suggest that the heterogeneity of the tumor microenvironment after tumor formation in the SCID mouse could affect the changes in many genes, influencing resistance in breast cancer (Scheme 1). Among such components, the nervous system seems to be affected, as seen by the list of genes above mentioned. However, such a factor has been underestimated in clinical outcomes. Identifying neuronal signatures associated with treatment response will help to characterize neuronal influence on tumor progression and identify new treatment targets. The search for hormonotherapy-predictive biomarkers has been analyzed on merged transcriptomic datasets from public databases to obtain potential gene signatures.

Authors [46] presented prognostic and predictive signatures pertaining to the neurological system. Such a work indicated that through a better understanding of the molecular processes behind these genes, new prognostic biomarkers for prognosis prediction or chemotherapy sensitivity could be developed. They suggested that the generated signatures showed prognostic and predictive potential after analysis using Cox models. The signatures generated from the first and external cohorts were compared to 14 other published signatures. As a result, the T pipeline produced the 24 and 97 gene signatures (NervSign24 and NervSign97) for the nervous system. Such signals were not chemotherapy predictive, but they were predictive for prognosis and hormone therapy. NervSign97 and NervSign24 were the two top performers when their predictive performance was compared to 14 documented risk signatures in six hormonotherapy-treated groups. Brain neural progenitor presence and perineural invasion were found to be nervous system-related mechanisms that were positively associated with NervSign97 and a poor clinical prognosis in patients receiving hormone therapy, according to pathway enrichment scores and deconvolution analysis. Further investigation into the role of neuronal components in tumor progression is warranted in light of the identification of two nervous-system-related signatures via transcriptomic profiling that were verified in clinical samples as hormonotherapy-predictive signatures [46]. Such a study provided two hormonotherapy-predictive and prognostic signatures related to the nervous system. It can highlight tumor neuronal components as potential new targets for breast cancer therapy. Then, the development of personalized medicine could be the future of cancer therapy.

Another factor to be considered for the many genes affected in the T2 cell line is the innervation, another hallmark of cancer, that also includes neurons and not only fibroblasts, adipocytes, endothelial cells, and lymphocytes [47]. The neurons are now considered important in breast cancer progression.

The T2 cell line is derived from a solid tumor formed by factors present in the microenvironment; as nerve fibers usually originate from the peripheral nervous system [48,49]. Many cancers, including breast cancer [50,51], can progress due to tumor innervation [52]. It has been shown that innervation is linked to a poor prognosis (overall and disease-free survival) and that nerves stimulate the carcinogenesis of breast cancer [53]. Sympathetic, parasympathetic, and sensory nerves comprise the autonomic nervous system, which is the primary source of innervation for tumors. It is commonly recognized that, in physiological settings, the sympathetic and parasympathetic/vagal nerves regulate the activity of practically every organ [54].

The SLIT and ROBO signaling pathways regulate axon guidance, neuronal migration, and axonal remnants in the nervous system by acting as a neural targeting factor. Recent studies have shown that distinct tumor cells display varying expression patterns at different stages of tumor angiogenesis, cell invasion, metastasis, and infiltration, in addition to varying levels of SLIT/ROBO signaling. The SLIT and ROBO axon-guidance molecules are now known to perform new functions in the onset of liver fibrosis and cancer [55]. ROBO1 has been implicated as a tumor suppressor for various types of cancer, including breast cancer, and the transmembrane proline-rich γ-carboxyglutamic acid protein 4 (PRRG4) may be a novel target for treating metastatic breast cancer; however, the regulation of ROBO1 expression during tumorigenesis remains poorly understood. However, it was found that via downregulating Robo1, PRRG4 promoted breast cancer metastasis; thus, PRRG4 expression was found to be higher in breast tumors than in normal breast tissues. PRRG4 was found to boost metastasis in an experimental metastasis model and to promote the migration and invasion of breast cancer cells in experiments including PRRG4 overexpression and knockdown [56].

## 5. Conclusions

The study described in this article involves genes related to the nervous system and changes due to the effect of estrogen and radiation. The expression of genes related to the nervous system, including *EFNA1*, *ROBO1*, *KLK6*, *LIMK2*, *KLK7*, *NLGN*4X, *MBP*, and *NEDD9* was higher in the tumorigenic T2 than in the control cell line. There was a positive correlation between *ESR1* and *ROBO1* gene expression in BRCA-Basal patients, and *EGFR* showed a positive correlation with *LIMK2*, *ROBO1*, *NEDD9*, and *MBP* expression levels in BRCA-Basal patients, as well, demonstrating to be a good marker for carcinogenicity, especially for Basal breast cancer patients. *ROBO1*, *KLK6*, *LIMK2*, *KLK7*, *NLGN4X*, *MBP*, and *NEDD9* expression levels were significantly higher in normal tissues than in tumors, demonstrating the possibility of being a tumor suppressor gene. Thus, experimental investigations need to be focused on the connection between the central nervous system and cancer to comprehend the molecular processes required to create new prognostic biomarkers.

## Figures and Tables

**Table 1:** Gene selection raw data from the Affymetrix array U133A.

Probe ID	Ct/E	Ct/A3	E/A5	A3/A5	A5/T2	A3/T2	Gene Symbol
202023_at	1.2	−6.2	−1.4	5.2	−1.0	5.1	*EFNA1*
213194_at	1.2	−30.7	1.1	10.9	1.1	44	*ROBO1*
204733_at	−1.4	−6.9	−3.0	1.7	7.4	12.3	*KLK6*
210582_s	−1.2	−1.4	−1.2	−1.0	3.7	3.7	*LIMK2*
205778_at	−2.0	−59.4	−4.8	6.2	11.3	69.5	*KLK7*
221933_at	2.4	−21.8	−11.8	4.5	29.6	134.1	*NLGN4X*
210136_at	2.2	−2.2	−1.3	3.7	2.2	8.0	*MBP*
202150_s_at	1.1	−6.8	1.6	11.7	−3.7	3.2	*NEDD9*

C/E: MCF-10F/estrogen; C/A3: MCF-10F/Alpha3; E/A5: estrogen/Alpha5; A3/A5: Alpha3/Alpha5; A5/T2: Alpha5/Tumor2; A3/T2: Alpha3/Tumor2.

**Table 2:** Clinical relevance of gene expressions across various cancer types analyzed according to the disease stage factor.

Breast Cancer	*EFNA1*	*ROBO1*	*KLK6*	*LIMK2*	*KLK7*	*NLGN4X*	*MBP*	*NEDD9*
BRCA (*n* = 1100)	3, 4 ***	3, 4 ***	3, 4 ***	3, 4 ***	3, 4 ***	3, 4 ***	3, 4 ***	3, 4 ***
BRCA-Basal (*n* = 191)	N.S.	N.S.	N.S.	N.S.	N.S.	N.S.	N.S.	N.S.
BRCA-Her2 (*n* = 82)	4 *	4 **	4 **	4 **	4 *	4 **	4 *	4 *
BRCA-LumA (*n* = 568)	4 ***	4 ***	4 ***	4 ***	4 ***	4 ***	4 ***	4 ***
BRCA-LumB (*n* = 219)	4 **	4 **	4 **	4 **	4 **	4 *	4 *	4 **

The statistical significance is annotated via the number of stars (*: *p* < 0.05; **: *p* < 0.01; ***: *p* < 0.001); 3, 4: clinical stage factor; N.S.: not significant. Data were estimated using TIMER2.0 in breast invasive carcinoma [30].

## Data Availability

TIMER2.0 is freely available at http://timer.cistrome.org (accessed on 6 August 2021); UCSC Xena online exploration tools are freely available at http://xena.ucsc.edu/ (accessed on 20 August 2021). The data generated in the present study may be requested from the corresponding author.
